# Copper Radical Oxidases Contribute to Virulence in *Sclerotinia sclerotiorum*

**DOI:** 10.3390/pathogens15090885

**Published:** 2026-08-24

**Authors:** Jinyi Tan, Jessica Kalyun Fong, Harry Brumer, Xin Li

**Affiliations:** 1 Michael Smith Laboratories, University of British Columbia, Vancouver, BC V6T 1Z4, Canadajessica.fong@msl.ubc.ca (J.K.F.);; 2 Department of Botany, University of British Columbia, Vancouver, BC V6T 1Z4, Canada; 3 Department of Chemistry, University of British Columbia, Vancouver, BC V6T 1Z1, Canada

**Keywords:** plant fungal pathogen, *Sclerotinia sclerotiorum*, auxiliary activity family 5 copper radical oxidases, virulence, sclerotia

## Abstract

*Sclerotinia sclerotiorum* is a broad-spectrum soilborne fungal pathogen, causing substantial yield losses in crops worldwide. Auxiliary Activity Family 5 (AA5) copper radical oxidases (CROs) are members of the carbohydrate active enzymes (CAZymes), valued for their potential as biocatalysts. Although they were reported to contribute to fungal development and disease progression in some phytopathogenic fungi such as *Colletotrichum graminicola*, the roles of AA5 enzymes in *S. sclerotiorum* remain largely unexplored. Here, we systematically investigated *S. sclerotiorum* AA5 genes through reverse genetics and biochemical approaches. Phylogenetic analysis grouped the *S. sclerotiorum* AA5 proteins into two subfamilies, including three subfamily 1 (AA5_1) members and one subfamily 2 (AA5_2) member. Enzymatic characterization revealed that one AA5_1 enzyme, SsAA5c, exhibited the highest catalytic specificity towards D-glyceraldehyde compared with methylglyoxal and glycerol. In addition, a predicted peroxidase gene (*SsPX1*) was identified in tandem with the putative AA5_2 gene, *SsAA5d*. Phenotypic analysis of gene deletion mutants demonstrated that these genes differentially affect fungal development and virulence. While their exact physiological substrates await identification, our findings highlight important roles of AA5 oxidases and the associated peroxidase in the biology of *S. sclerotiorum*, providing insights that may contribute to future improved disease management strategies.

## 1. Introduction

*Sclerotinia sclerotiorum* is a soilborne phytopathogen, representing the Sclerotiniaceae family within the Ascomycota phylum [1,2]. It is recognized as one of the most aggressive necrotrophic pathogens, capable of infecting over 600 plant species and causing considerable yield losses in a wide range of economically important crops, including canola, vegetables such as lettuce and tomato, legumes such as soybean and dry bean, and sunflower [1,3,4,5]. It is present in nearly all major agricultural regions and has led to substantial economic impacts worldwide [6,7,8,9]. A key feature of this pathogen is its capacity to form sclerotia, dormant survival structures enclosed by melanized outer layers [10,11]. Sclerotia have dual roles: they function as survival structures that enable the fungus to persist through unfavorable conditions and also contribute to its reproductive cycle [2]. Sclerotia can germinate as mycelia that directly invade plant tissues. They can also germinate as apothecia that produce wind-dispersed ascospores, enabling the pathogen to infect surrounding host plants [2,12]. During infection, it forms compound appressoria to aid penetration of the host cell wall [2]. Due to its wide host range and the ability to produce long-lived sclerotia, controlling *S. sclerotiorum* diseases is especially difficult. Although it poses major agricultural challenges, the molecular mechanisms underlying how it perceives external signals, develops critical structures such as sclerotia, apothecia, and appressoria, and interacts with its plant hosts are still not well understood.

Many phytopathogenic fungi employ a range of cell wall-degrading enzymes (CWDEs) to degrade components of the plant cell wall to facilitate host entry [13,14]. CWDEs belong to the carbohydrate active enzyme (CAZyme) classification, which comprise enzyme classes such as glycoside hydrolases (GHs), polysaccharide lyases (PLs), carbohydrate esterases (CEs), glycosyl transferases (GTs) and auxiliary activities (AAs) [15,16]. From omics data, the *S. sclerotiorum* genome contains up to 529 CAZymes, where a large number of the encoded genes are induced during infection [17,18,19,20]. In particular, the AA class contains 18 families of diverse oxidoreductases. Within this class, family 5 (AA5) has recently garnered significant attention for their roles in fungal pathogenesis, which are not well understood as the majority of the literature on AA5 enzymes has focused on their industrial potential as biocatalysts [21,22].

Fungal AA5 members, also known as copper radical oxidases (CROs), are classified into two subfamilies, subfamily 1 (AA5_1) and subfamily 2 (AA5_2), which are primarily active on aldehydes (e.g., methylglyoxal) and alcohols (e.g., galactosides and aliphatic or aromatic alcohols), respectively [23]. The oxidation of substrates by AA5 enzymes is coupled to the concomitant production of hydrogen peroxide as a co-product [21,22,24,25]. Early studies on basidiomycete white-rot *Phanerochaete* fungi have implicated these enzymes in biomass degradation as accessory enzymes that generate hydrogen peroxide for ligninolytic enzymes [26,27]. Following this, several studies on other fungi, including both pathogenic and saprophytic species, have shown that AA5 enzymes participate in fungal morphogenesis and virulence [28,29,30,31,32]. For instance, in the human pathogenic yeast *Cryptococcus neoformans*, the secreted AA5 enzyme Glx1 is essential for infectivity, while the other related proteins Glx2 and Glx3 are not required for virulence [29]. Similarly, in the plant pathogen *Colletotrichum orbiculare*, an AlcOx enzyme and a peroxidase encoded by its neighboring gene facilitate host penetration and promote fungal infection [28]. In contrast, AA5 proteins make only a modest contribution to pathogenicity in the vascular wilt fungus *Verticillium dahliae* [33]. Despite these findings, the biological roles of AA5 members in *S. sclerotiorum* remain largely unclear.

In this study, we identified and characterized AA5 enzymes in *S. sclerotiorum*, which fall within the two canonical subfamilies. Biochemical analysis was successful for a single subfamily 1 enzyme, which exhibited the highest catalytic specificity toward D-glyceraldehyde. Furthermore, phenotypic analyses of targeted deletion mutants revealed that although these AA5 enzymes are largely dispensable for vegetative hyphal growth, specific members are necessary for proper sclerotia development and full virulence.

## 2. Materials and Methods

### 2.1. Fungal Strains and Culture Conditions

The wild-type (WT) strain *S. sclerotiorum* 1980 and all derived mutant strains were grown on potato dextrose agar (PDA, Shanghai Bio-way technology, Shanghai, China) at room temperature. Strains were stored either on PDA slants at 4 °C or in the form of sclerotia for long-term preservation. Knockout (KO) mutants of *S. sclerotiorum* were selected and purified on PDA supplemented with hygromycin B (50 μg/mL, Sigma, MilliporeSigma, Oakville, ON, Canada) to confirm successful transformation and selection.

### 2.2. Phylogenetic Analysis

Amino acid sequences of 252 AA5 members comprising only the catalytic modules were aligned with MAFFT and this alignment was used to generate a maximum likelihood (ML) phylogenetic tree. The Dayhoff substitution model was used for phylogenetic inference. Bioinformatic analyses were performed using tools available through the CIPRES Science Gateway. The ML tree was generated using RAxML-HPC2 (Cipres Science Gateway Online Server) with 100 bootstrap replicates. Protein sequences used for phylogenetic analysis were obtained from the CAZy (https://www.cazy.org/, accessed on 1 July 2026), GenBank (https://www.ncbi.nlm.nih.gov/genbank/, accessed on 1 July 2026), JGI MycoCosm (https://mycocosm.jgi.doe.gov/mycocosm/home, accessed on 1 July 2026), and Ensembl Fungi (https://fungi.ensembl.org, accessed on 1 July 2026) databases.

### 2.3. Cloning and Recombinant Strain Production

cDNAs encoding for the full-length constructs of the AA5 candidates were commercially synthesized (Thermo Fisher Scientific, Waltham, MA, USA), with their codons optimized. The sequences of the recombinant constructs are listed in Supplemental Table S1. These were cloned into the pPICZαA vector containing the *Saccharomyces cerevisiae* α-factor secretion signal using the EcoRI (New England Biolabs, Ipswich, MA, USA) and XbaI (New England Biolabs, Ipswich, MA, USA) restriction sites. Recombinant plasmids were transformed into competent *Escherichia coli* DH5α via heat shock and then purified from cultures containing low-salt LB medium with 25 µg/mL zeocin. To produce recombinant *Komagataella phaffii* (syn. Pichia pastoris), 5 µg of each purified plasmids was linearized with PmeI and transformed into yeast cells via electroporation. Transformed *K. phaffii* were plated onto YPD agar containing 100 or 500 µg/mL of zeocin for selection of successful transformants.

### 2.4. Protein Production and Purification

Shake flask protein production was performed as previously described [34]. Protein purification was carried out using immobilized metal affinity chromatography with subsequent buffer exchange as described [34]. Purified protein was analyzed on pre-cast SDS-PAGE polyacrylamide gels (4–20% *w*/*v*, BioRad, Hercules, CA, United States). Protein concentrations were measured by absorbance at 280 nm and extinction coefficients were calculated with the ProtParam tool on the ExPASY server (https://web.expasy.org/protparam/, accessed on 1 July 2026).

### 2.5. Activity and pH Screening

Enzyme activity on selected substrates was measured using the horseradish peroxidase (HRP, Bio Basic, Markham, ON, Canada) and 2,2-azinobis (3-ethylbenzthiazoline-6-sulfonic acid) (ABTS, Sigma-Aldrich, Burlington, MA, USA) coupled colorimetric assay (0.1 mg/mL HRP, 0.25 mg/mL ABTS) in a 200 µL reaction volume (50 mM sodium phosphate, buffer pH 7.0). Activity was measured by following changes in absorbance at 415 nm using a BioTek Epoch spectrophotometer (Agilent Technologies, Santa Clara, CA, USA). Aldehydes were assayed at 10 mM; galactose and glycerol were assayed at 300 mM. All measurements were carried out in triplicate at ambient temperature (ca. 21 °C). Enzyme activity over a range of pH values was measured as described [34] using the HRP-ABTS coupled assay.

### 2.6. Michaelis–Menten Kinetics

Selected substrates were assayed across a range of concentrations to determine the Michaelis–Menten parameters. Activity was measured using the HRP-ABTS coupled assay (0.46 mM ABTS, 21 U/mL HRP in 50mM sodium phosphate buffer) at ambient temperature (ca. 21 °C) and at the optimal pH of 9.0. Measurements were carried out in triplicate with a 1 mL reaction volume and absorbance at 415 nm was measured on a CARY 60 UV-Vis spectrophotometer. The Michaelis–Menten equation was fitted to the data with OriginPro (Originlab 9.85), and in cases where enzyme saturation was not reached, a linear fit of the data was applied.

### 2.7. Target Gene Knockout (KO)

Gene deletion mutants were generated through a homologous recombination-based method as described by Xu et al. [35]. Briefly, actively growing WT *S. sclerotiorum* mycelia were enzymatically digested in Novozyme buffer containing VinoTaste Pro cell wall digestion enzyme (Novozymes, Vancouver, BC, Canada) to produce protoplasts. The knockout cassette, comprising the 5′ and 3′ flanking sequences of the target locus fused to a hygromycin resistance marker (*HYG*), was introduced into WT protoplasts using polyethylene glycol (PEG)-mediated transformation.

Transformants exhibiting resistance to hygromycin B were selected on PDA amended with hygromycin B. On-target marker insertion was confirmed via colony PCR using junction-specific primer pairs (7F/H855R and H855F/8R) (Thermo Fisher Scientific, Waltham, MA, USA). Positive transformants were purified by successive rounds of hyphal-tip isolation and protoplast regeneration. This purification process was repeated until PCR with primer pair 5F/6R confirmed the complete absence of the WT allele, demonstrating successful target gene deletion. Primer sequences used in this study are listed in Supplemental Table S2.

### 2.8. S. Sclerotiorum Growth Rate and Colony Morphology Assessment

Mycelial plugs (5 mm diameter) were excised from the actively expanding margins of WT and mutant colonies using a sterile pipette tip and placed at the center of fresh PDA plates (85 mm diameter). Plates were incubated at room temperature, with three independent biological replicates per strain. Colony diameters were recorded at 12 h intervals until the mycelia extended to the edge of the plates. Growth rates during the linear growth phase (0–36 h) were calculated by linear regression and compared using an extra sum-of-squares F-test.

Colony morphology was documented at 7 days post inoculation to evaluate sclerotium development. The number of sclerotia was determined on PDA plates after 14 days of growth, using three biological replicates per strain. Sclerotia were then harvested and imaged. Sclerotium size was measured using ImageJ software (1.53v) for quantitative analysis. The WT control was included in each experiment and cultured concurrently with the corresponding mutant strains. As the same WT samples served as the control for multiple mutant analyses, identical representative WT images are presented across the relevant figures for clarity and consistency.

### 2.9. Plant Infection Assay

Actively growing mycelial plugs (1 mm or 5 mm in diameter) were excised and inoculated onto detached leaves of 3–4-week-old *Arabidopsis thaliana* (Col-0) or 4–5-week-old *Nicotiana benthamiana* plants, respectively. Inoculated leaves were arranged on moist paper towels on a covered tray to maintain humidity. The trays were incubated in a growth chamber (23 °C; 16 h light/8 h dark). Disease progression was evaluated by measuring lesion areas using ImageJ software (1.53v) for quantitative analysis.

### 2.10. Compound Appressoria Observation

Fresh mycelial plugs (5 mm in diameter) were taken from the actively growing edge of colonies and placed onto glass slides. The slides were incubated in Petri dishes lined with moist paper towels at room temperature for 2 days. The development of compound appressoria was examined and recorded using a ZEISS light microscope (Jena, Germany). The length of compound appressoria branches was measured using ImageJ (1.53v) for quantitative analysis. The WT control was included in each experiment and cultured concurrently with the corresponding mutant strains. As the same WT samples served as the control for multiple mutant analyses, identical representative WT images are presented across the relevant figures for clarity and consistency.

### 2.11. Statistical Analysis

The statistical significance of the data was determined using GraphPad Prism 10 software (GraphPad Software Inc., San Diego, CA, USA: http://www.graphpad.com/, accessed on 1 July 2026). Data were analyzed by one-way ANOVA followed by Tukey’s multiple comparison test. Statistical significance was represented by distinct lettering in the figures. A significance threshold of *p* < 0.05 was used as the default criterion, while a more stringent threshold of *p* < 0.01 was applied for specific analyses as indicated in the corresponding figure legends.

## 3. Results

### 3.1. Identification and Classification of Copper Radical Oxidases (CROs) from Auxiliary Activity Family 5 (AA5) in S. sclerotiorum

**Figure 1:**
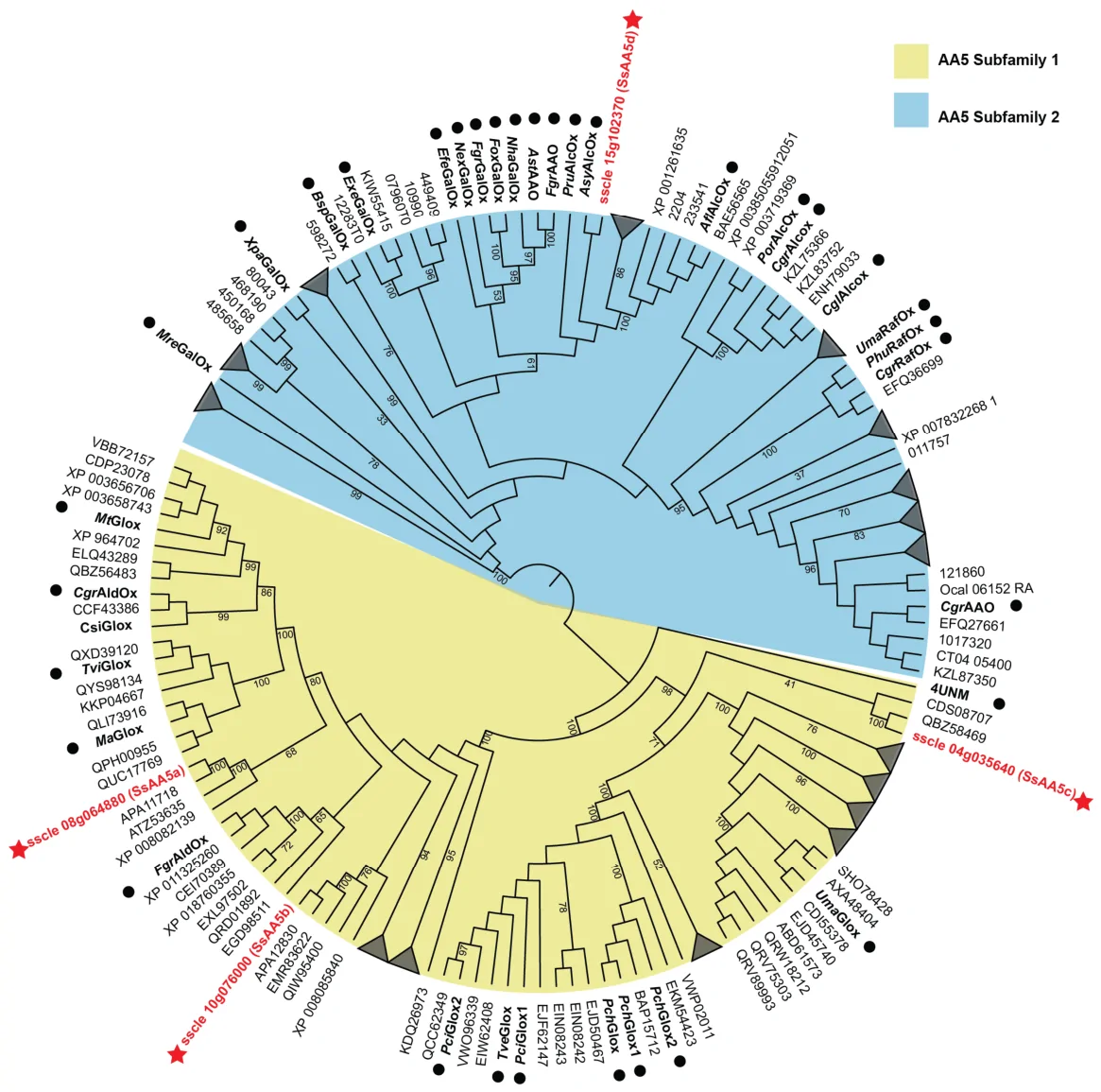
Candidate Auxiliary Activity Family 5 (AA5) genes from *Sclerotinia sclerotiorum*. Candidate genes from *S. sclerotiorum* are highlighted in red and denoted with stars. Bootstrap values are presented at nodes to support the sequence groups. Reported members from AA5 subfamilies 1 and 2 (yellow and blue, respectively) are in bold text and denoted by circles. Clades containing uncharacterized AA5 members are shown as triangles to reduce the visual complexity of the phylogeny. Abbreviations are as follows: GalOx = galactose oxidase, AlcOx = alcohol oxidases, AAO = aryl alcohol oxidases, Mre = *Mytilinidion resinicola*, Xpa = *Xanthoria parietina*, Bsp = *Bisporella* spp., Exe = *Exophialia xenobiotica*, Efe = *Epichloe festucae*, Nex = *Niesslia exilis,* Fgr = *Fusarium graminearum,* Fox = *Fusarium oxysporum,* Nha = *Nectria haematococca,* Ast = *Acremonium strictum,* Pru = *Penicillium rubens,* Asy = *Aspergillus sydowii,* Afl = *Aspergillus flavus,* Por = *Pyricularia oryzae,* Cgr = *Colletotrichum graminicola,* Cgl = *Colletotrichum gloeosporioides,* Uma = *Ustilago maydis,* Phu = *Pseudozyma hubeiensis,* Pch = *Phanerochaete chrysosporium,* Pci = *Pycnoporus cinnabarinus,* Tve = *Trametes versicolor,* Ma = *Metarhizium acridum,* Tvi = *Trichoderma virens,* Mt = *Myceliophthora thermophila*.

Using protein BLAST (https://blast.ncbi.nlm.nih.gov/Blast.cgi; https://fungi.ensembl.org/Multi/Tools/Blast, accessed on 1 July 2026), four putative copper radical oxidases (CROs) belonging to Auxiliary Activity Family 5 (AA5) were identified in *S. sclerotiorum*, all of which are encoded by single-copy genes (Figure 1). Three enzymes encoded by the genes *sscle_08g064880* (*SsAA5a*), *sscle_10g076000* (*SsAA5b*), and *sscle_04g035640* (*SsAA5c*) were classified within AA5 subfamily 1 (AA5_1), showing 45.46%, 45.95%, and 39.85% sequence similarity, respectively, to the characterized AA5_1 enzyme FgrAldOx from *Fusarium graminearum* [36]. These three proteins are predicted to be secreted based on N-terminal signal peptide analysis (Figure S1A). Protein sequence alignment analysis indicates that both SsAA5a and SsAA5b contain the wall stress-responsive component (WSC) domain, which characteristically includes four conserved disulfide bridges (eight cysteine residues; Figure S1A,B). In contrast, *sscle_15g102370* (*SsAA5d*) was assigned to AA5 subfamily 2 (AA5_2) and exhibited 51.57% sequence similarity to the well-characterized AA5_2 enzyme FgrGalOx from *F. graminearum* (Figure 1) [21].

### 3.2. A S. sclerotiorum AA5 Subfamily 1 Enzyme Shows Preference for D-Glyceraldehyde Oxidation

**Figure 2:**
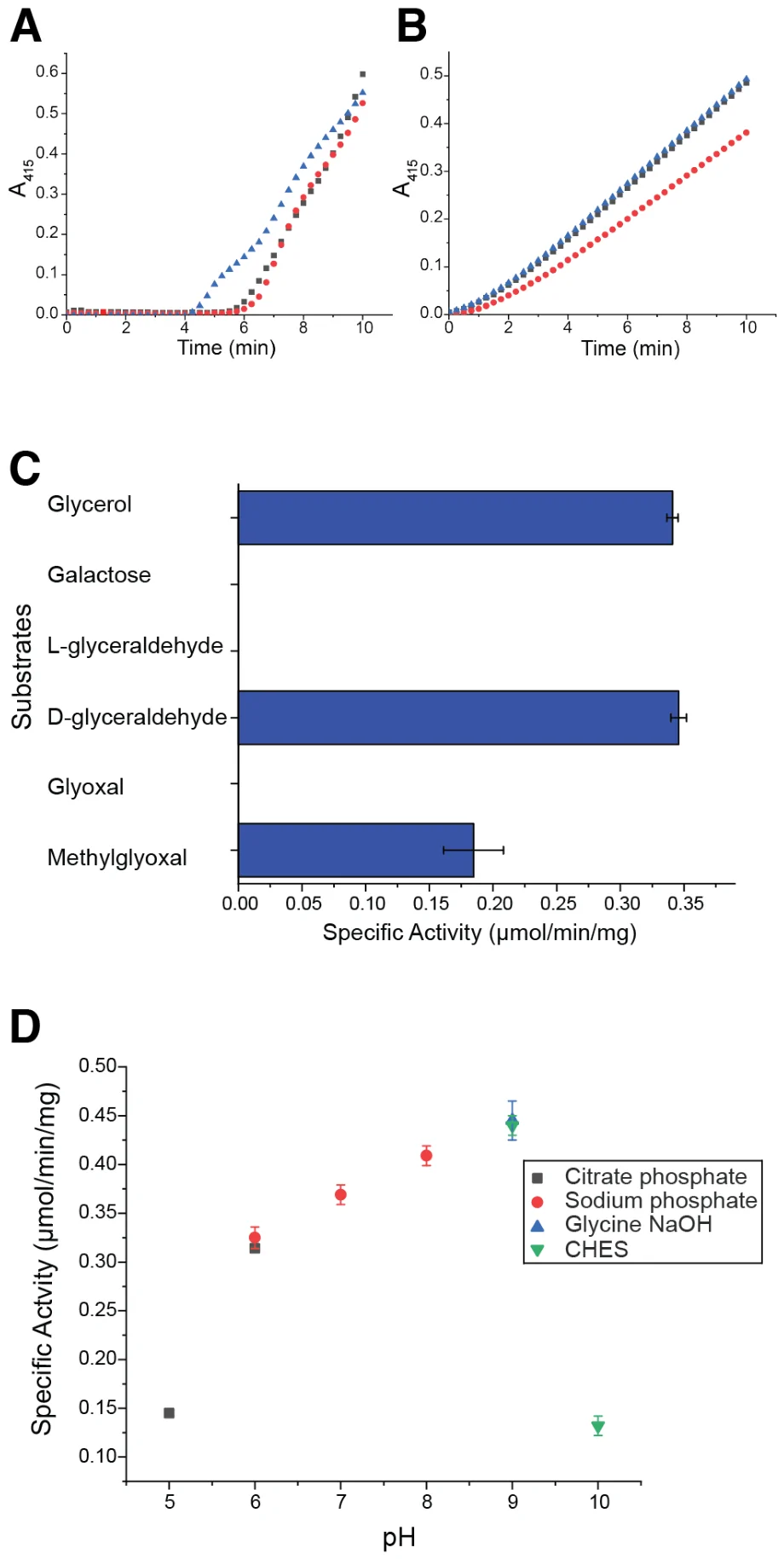
Biochemical characterization of one AA5 subfamily 1 (SsAA5c) enzyme. (**A**,**B**) Preliminary activity screen on 10 mM methylglyoxal and purified SsAA5c. (**A**) Activity trace with purified enzyme (0.2 mg/mL) that was not treated with potassium ferricyanide. (**B**) Activity trace with purified enzyme after 10 min treatment with 10 mM potassium ferricyanide (0.1 mg/mL enzyme). The three lines in each panel represent three independent replicates. (**C**) Activity screen of SsAA5c on a small panel of common AA5_1 substrates. Aldehydes were screened at 10mM, and galactose and glycerol at 300 mM. Substrates were screened in triplicate at room temperature in 96-well-plate format in 50 mM sodium phosphate buffer, pH 7.0, with the HRP-ABTS coupled assay. (**D**) pH rate profile of SsAA5c. A range of buffers at 50 mM final concentration were screened. Activity was measured on 300 mM glycerol.

Candidate AA5 members from subfamilies 1 and 2 were selected for recombinant expression and subsequent biochemical characterization. Full-length sequences without signal peptide sequences were cloned into *Komagataella phaffii* (syn. Pichia pastoris. X-33). However, only one construct (sscle_04g035640, SsAA5c) was successfully produced. Interestingly, long lag times of over five minutes were seen during initial activity screens, indicating a slow activation of the enzyme by the horseradish peroxidase in the assay components. As such, the enzyme was treated with potassium ferricyanide to oxidatively activate it before assaying activity (Figure 2A,B). All subsequent activity assays were performed with pre-activated enzymes. Preliminary activity screens on a panel of representative substrates (Figure 2C) revealed an activity profile similar to those of previously characterized AA5_1 members, where small aldehydes such as glyceraldehyde and methylglyoxal are preferentially oxidized over primary alcohols or carbohydrates [36,37,38,39]. Similar to some recently characterized AA5_1 enzymes, enantioselective preference for D-glyceraldehyde over the L-isomer was also observed [36]. For more detailed analysis of enzyme specificity, initial rate kinetics were measured at ambient temperature at the optimal pH 9.0 (Figure 2D) for the substrates with the best activity. Michaelis–Menten parameters (Table 1) show that the enzyme displayed the highest catalytic specificity for D-glyceraldehyde compared to methylglyoxal or glycerol (Figure S2).

### 3.3. AA5_1 Members Contribute to Virulence in S. sclerotiorum

**Figure 3:**
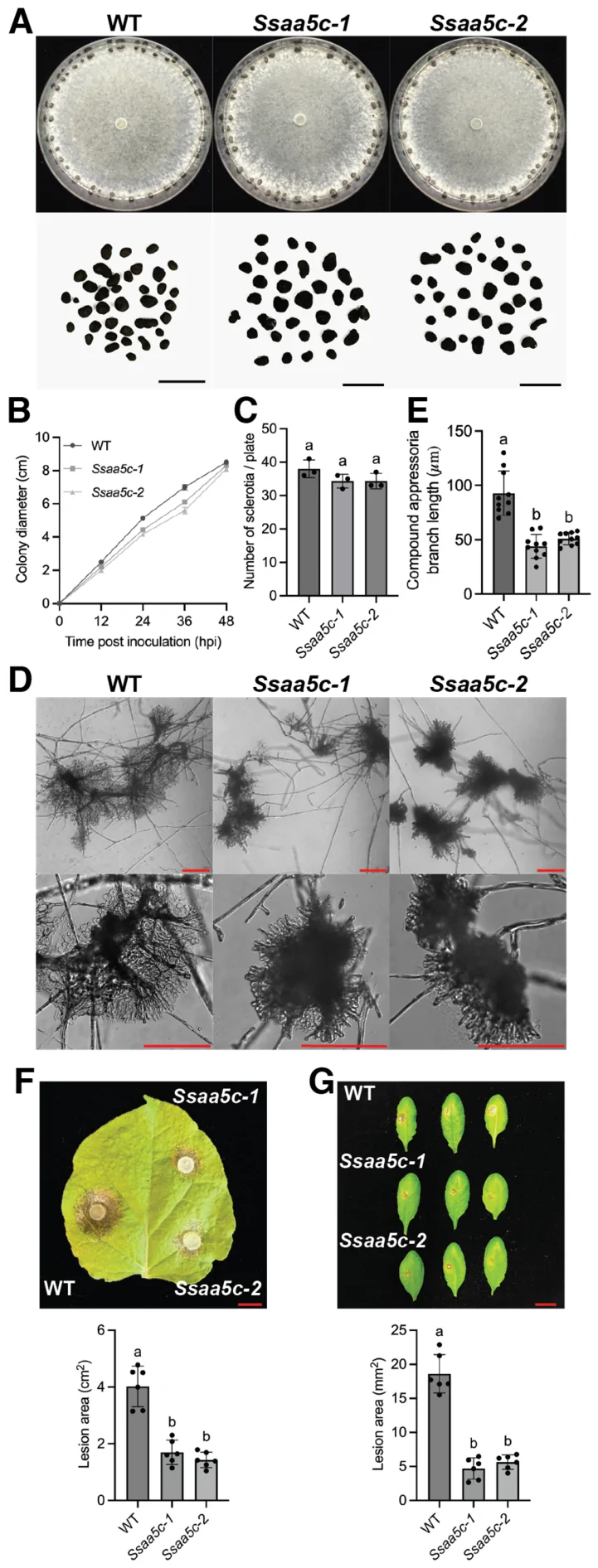
Phenotypic characterization of AA5_1 *Ssaa5c* knockout mutants. (**A**) Colony morphology and sclerotia development of the WT and *Ssaa5c* knockout (KO) mutants. Images were captured at 10 days post inoculation (dpi). Sclerotia were harvested from cultures grown for 14 days. Scale bar = 1 cm. (**B**) Radial growth of WT and *Ssaa5c* KO mutants over a 48 h period. Colony diameters were measured every 12 h. Data are presented as the mean ± standard deviation (SD) from three biological replicates. (**C**) Quantification of sclerotia formation in WT and *Ssaa5c* KO mutants based on three independent trials. (**D**) Microscopic observation of compound appressoria produced by WT and *Ssaa5c* KO mutants on glass slides following transfer from PDA cultures. Images were obtained at 2 dpi. Scale bar = 200 μm. (**E**) Quantification of compound appressoria branch length (*n* = 10). (**F**) Virulence of *Ssaa5c* KO mutants on intact *N. benthamiana* leaves. Representative lesion phenotypes at 36 h post inoculation (hpi) are shown in the upper panel, with corresponding lesion area measurements presented in the lower panel (*n* = 6). Scale bar = 1 cm. Each dot represents an individual lesion measurement. Different letters indicate statistically significant differences (*p* < 0.01). Three independent experiments were performed with similar results. (**G**) Virulence of *Ssaa5c* KO mutants on intact *A. thaliana* leaves. Representative lesion phenotypes at 48 hpi are shown in the upper panel, with corresponding lesion area measurements presented in the lower panel (*n* = 6). Scale bar = 10 mm. Each dot represents an individual lesion measurement. Different letters indicate statistically significant differences (*p* < 0.01). Three independent experiments were performed with similar results.

**Figure 4:**
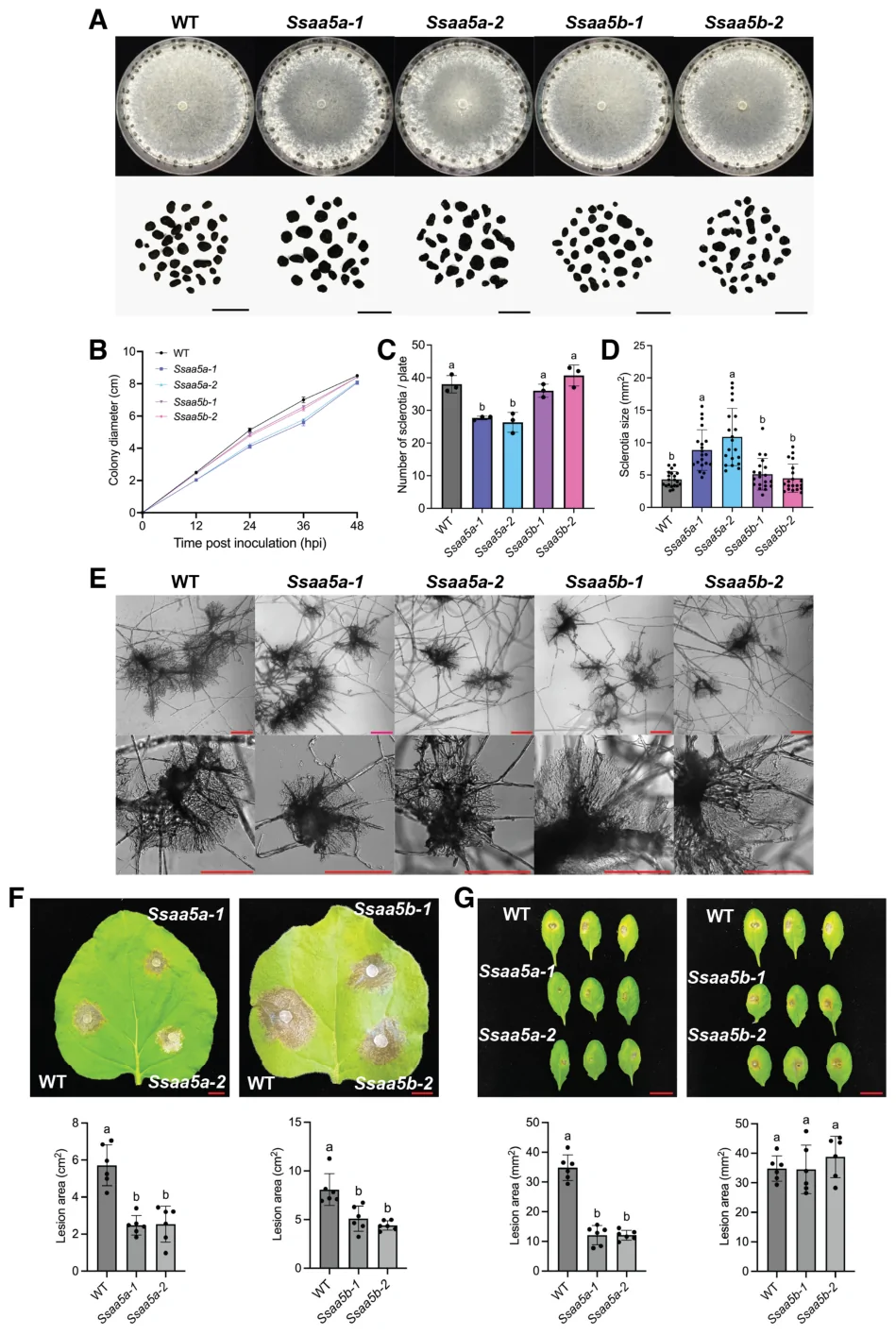
Phenotypic characterization of AA5_1 (*Ssaa5a* and *Ssaa5b*) knockout mutants. (**A**) Colony morphology and sclerotium development of the WT, *Ssaa5a* and *Ssaa5b* KO mutants. Images were captured at 10 days post inoculation (dpi). Sclerotia were harvested from cultures grown for 14 days. Scale bar = 1 cm. (**B**) Radial growth of WT, *Ssaa5a* and *Ssaa5b* KO mutants over a 48 h period. Colony diameters were measured every 12 h. Data are presented as the mean ± standard deviation (SD) from three biological replicates. The *Ssaa5a* mutants exhibited a significantly reduced growth rate compared with the WT (*p* = 0.0315). (**C**) Quantification of sclerotia formation in WT, *Ssaa5a* and *Ssaa5b* KO mutants based on three independent trials. (**D**) Quantification of sclerotia size in WT, *Ssaa5a* and *Ssaa5b* KO mutants (*n* = 20). (**E**) Microscopic observation of compound appressoria produced by WT, *Ssaa5a* and *Ssaa5b* KO mutants on glass slides following transfer from PDA cultures. Images were obtained at 2 dpi. Scale bar = 200 μm. (**F**) Virulence of *Ssaa5a* and *Ssaa5b* KO mutants on intact *N. benthamiana* leaves. Representative lesion phenotypes at 36 h post inoculation (hpi) are shown in the upper panel, with corresponding lesion area measurements presented in the lower panel (*n* = 6). Scale bar = 1 cm. Each dot represents an individual lesion measurement. Different letters indicate statistically significant differences (*p* < 0.01). Three independent experiments were performed with similar results. (**G**) Virulence of *Ssaa5a* and *Ssaa5b* KO mutants on intact *A. thaliana* leaves. Representative lesion phenotypes at 48 hpi are shown in the upper panel, with corresponding lesion area measurements presented in the lower panel (*n* = 6). Scale bar = 10 mm. Each dot represents an individual lesion measurement. Different letters indicate statistically significant differences (*p* < 0.01). Three independent experiments were performed with similar results.

To investigate the roles of these AA5 genes in *S. sclerotiorum*, single-gene-deletion mutants were generated using a homologous recombination strategy (Figure S3A). Two independent knockout (KO) mutants of *SsAA5c* were successfully obtained (Figure S3B). These mutants showed normal sclerotia development compared with the wild type (WT) (Figure 3A–C). Interestingly, their compound appressoria exhibit shorter branches and the mutants caused smaller lesion sizes on both *Nicotiana benthamiana* and *Arabidopsis thaliana* leaves, suggesting critical roles of *SsAA5c* in infection structure development and virulence (Figure 3D–G). Deletion of *SsAA5a* resulted in the formation of fewer but larger sclerotia, and a slightly reduced growth rate (Figure 4A–D). Although compound appressoria formed normally, the mutants exhibited reduced virulence on different plant hosts (Figure 4E–G). In contrast, the *Ssaa5b* KO mutants are morphologically similar to the WT, with no defects in compound appressoria formation, but caused smaller lesions on *N. benthamiana* leaves (Figure 4). These results indicate that members of AA5 subfamily 1 play important yet varying roles in the development and virulence of *S. sclerotiorum*.

### 3.4. AA5_2 Member (SsAA5d) and the Peroxidase (SsPX1) Are Also Involved in the Full Virulence of S. sclerotiorum

**Figure 5:**
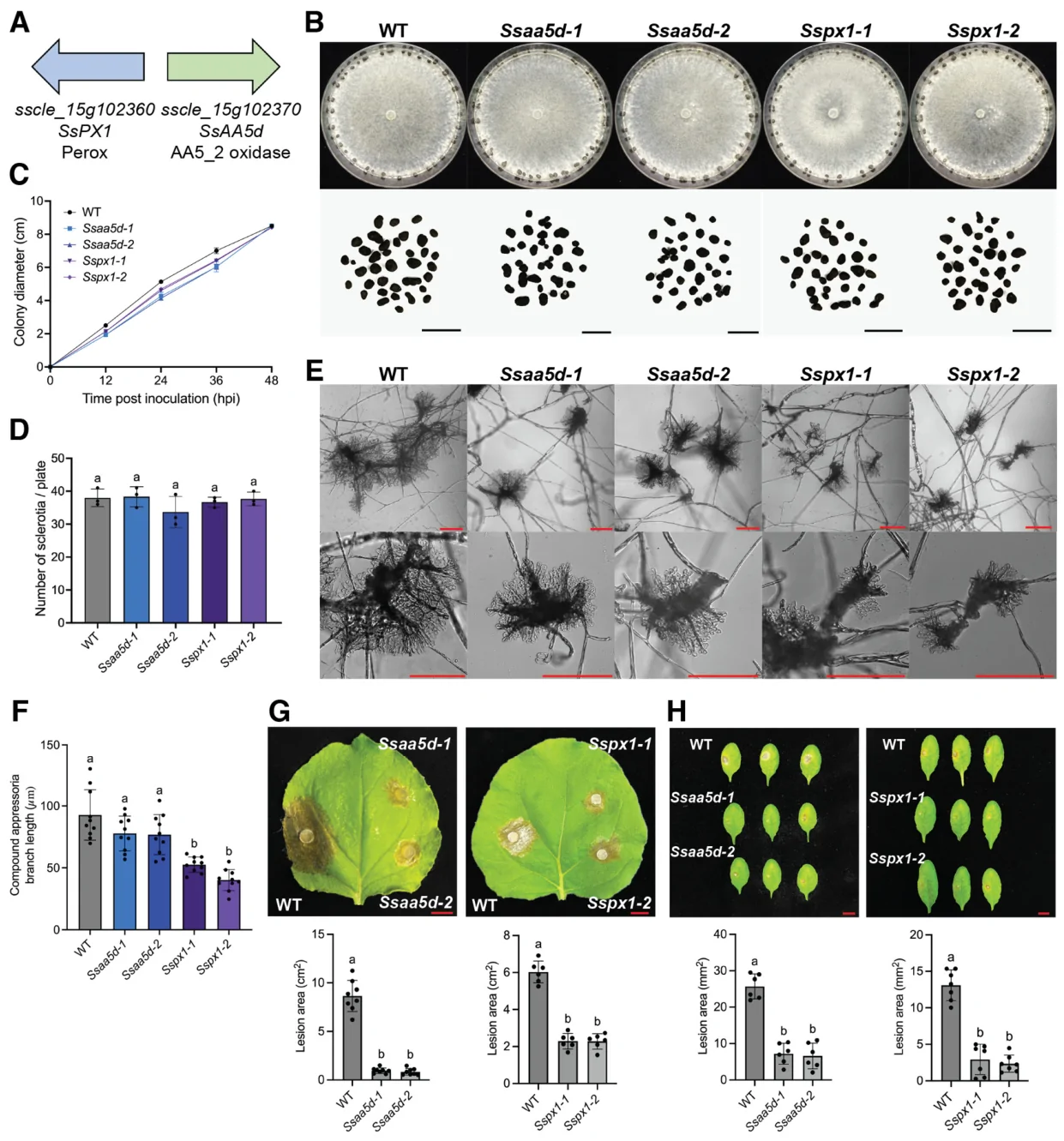
Phenotypic characterization of AA5 subfamily 2 (*SsAA5d*) and peroxidase (*SsPX1*) knockout mutants. (**A**) Schematic representation of the putative tandem oxidase system in *S. sclerotiorum*. (**B**) Colony morphology and sclerotia development of the WT, *Ssaa5d* and *Sspx1* KO mutants. Images were captured at 10 days post inoculation (dpi). Sclerotia were harvested from cultures grown for 14 days. Scale bar = 1 cm. (**C**) Radial growth of WT, *Ssaa5d* and *Sspx1* KO mutants over a 48 h period. Colony diameters were measured every 12 h. Data are presented as the mean ± standard deviation (SD) from three biological replicates. (**D**) Quantification of sclerotia formation in WT, *Ssaa5d* and *Sspx1* KO mutants based on three independent trials. (**E**) Microscopic observation of compound appressoria produced by WT, *Ssaa5d* and *Sspx1* KO mutants on glass slides following transfer from PDA cultures. Images were obtained at 2 dpi. Scale bar = 200 μm. (**F**). Quantification of compound appressoria branch length (*n* = 10). (**G**) Virulence of *Ssaa5d* and *Sspx1* KO mutants on intact *N. benthamiana* leaves. Representative lesion phenotypes at 36 h post inoculation (hpi) are shown in the upper panel, with corresponding lesion area measurements presented in the lower panel (*n* = 6). Scale bar = 1 cm. Each dot represents an individual lesion measurement. Different letters indicate statistically significant differences (*p* < 0.01). Three independent experiments were performed with similar results. (**H**) Virulence of *Ssaa5d* and *Sspx1* KO mutants on intact *A. thaliana* leaves. Representative lesion phenotypes at 48 hpi are shown in the upper panel, with corresponding lesion area measurements presented in the lower panel (*n* = 6). Scale bar = 10 mm. Each dot represents an individual lesion measurement. Different letters indicate statistically significant differences (*p* < 0.01). Three independent experiments were performed with similar results.

Two independent single-gene-deletion mutants of *Ssaa5d*, the subfamily 2 member, were also generated. These mutants displayed normal fungal development but reduced virulence on different plant hosts (Figure 5). Interestingly, a predicted peroxidase gene (*sscle_15g102360*, *SsPX1*) was identified clustering in tandem with a putative oxidase gene *sscle_15g102370* (*SsAA5d*) (Figure 5A). Similarly, deletion of this peroxidase did not alter fungal vegetative growth and sclerotia formation (Figure 5B–D), but resulted in defective compound appressoria characterized by shorter branches and reduced virulence (Figure 5E–H). Taken together, these results indicate that members of the AA5 enzymes and their associated peroxidase play critical yet varying roles in development and virulence of *S. sclerotiorum*.

## 4. Discussion

Plant pathogens represent a major threat to global food security. To successfully infect their hosts, they secrete a diverse repertoire of cell wall-degrading enzymes (CWDEs) that facilitate penetration of the plant surface and subsequent tissue colonization. CWDEs are a functionally specialized subset of carbohydrate active enzymes (CAZymes), which include the auxiliary activity (AA) class. Within the AA class, family 5 (AA5) is particularly interesting due to its roles in diverse fungal pathogens. Although AA5 enzymes have been studied in some fungal species, their biochemical mechanisms and biological functions in *S. sclerotiorum* remain poorly understood. In this study, we identified and functionally characterized AA5 enzymes in *S. sclerotiorum*, including three members belonging to subfamily AA5_1 and one enzyme from subfamily AA5_2. An associated peroxidase was also genetically characterized.

Reverse genetic analyses revealed three AA5_1 members (SsAA5a, SsAA5b, and SsAA5c) in *S. sclerotiorum*, all of which are predicted secreted proteins containing N-terminal signal peptides. Among them SsAA5a and SsAA5b possess wall stress-responsive component (WSC) domains. Phylogenetic analysis further showed that these two enzymes cluster within a distinct monophyletic group, separate from SsAA5c. These findings are consistent with previous reports on *F. graminearum* and *C. graminicola* [36]. Biochemical profile characterization of SsAA5c revealed its strong preference for small aldehydes, particularly D-glyceraldehyde, over primary alcohols and carbohydrate substrates. The observed specificity is similar to previously characterized AA5_1 enzymes in other fungal species, indicating a conserved enzymatic role within this family, although differences in substrate preference may reflect species-specific adaptation [36,37,38,39].

In addition, single-gene-deletion mutants were generated for all three AA5_1 genes. Although complementation strains were not constructed to definitively rule out potential off-target effects or secondary mutations, all phenotypic assays were conducted using two independent deletion alleles per targeted gene to mitigate this constraint. The consistent phenotypes observed across independent lines reinforce the causal relationship between the deletions and the mutant phenotypes. Phenotypic analyses revealed that these enzymes are largely dispensable for *S. sclerotiorum* vegetative development, with the exception of SsAA5a, which is involved in proper sclerotia development. A comparable phenotype has been reported in the hemi-biotrophic phytopathogenic fungus *Colletotrichum siamense*, in which an AA5_1 protein containing a signal peptide and five WSC domains is required for normal conidiation [32]. Although the AA5_1 enzymes are not essential for fungal growth, they contribute differentially to full virulence of *S. sclerotiorum*, suggesting that their functions are more closely associated with host infection than with core developmental processes. Interestingly, the *SsAA5b* deletion mutants displayed a host-dependent reduction in virulence, exhibiting significantly impaired lesion expansion on *N. benthamiana* while maintaining WT infection levels on *A. thaliana*. This contrast indicates that *SsAA5b* contributes to *S. sclerotiorum* pathogenicity in a host-specific manner, likely interacting with distinct host factors or defense mechanisms. Future comparative transcriptomic and metabolomic analyses across varied host species will help elucidate the mechanisms underlying this host-dependent virulence. In parallel, SsAA5c contributes to proper formation of compound appressoria, highlighting its specific role in the development of infection structures. This observation is consistent with studies in other fungal species, including *C. siamense*, *C. orbiculare*, *Ustilago maydis* and *Trichoderma virens*, where AA5_1 enzymes have been implicated in infection-related development and fungal pathogenicity [28,30,31,32].

One AA5_2 member, SsAA5d, was identified from reverse genetic analysis. Interestingly, a putative peroxidase gene (*SsPX1*) was found immediately adjacent to *SsAA5d*, forming a tandem peroxidase–oxidase gene pair. Similar genomic arrangements have been reported in several pathogenic ascomycetes [28]. However, organization of this gene pair varies among fungal species. In *Verticillium* species, the peroxidase and oxidase genes are arranged in a tail-to-tail orientation, whereas in *S. sclerotiorum*, they occur in a head-to-head configuration, consistent with patterns reported in *Colletotrichum* species and *Magnaporthe oryzae* [28,33,40]. In *C. orbiculare*, the Perox–AlcOx metalloenzyme pairs are specifically recruited during the early stages of penetration peg development and accumulate at the pore region formed between the fungus and the host cell [28]. Their activity on long-chain alcohols is proposed to trigger a mechanistically unresolved biochemical cascade that facilitates penetration and ultimately contributes to fungal pathogenicity [28].

Here, the biological functions of SsAA5d and SsPX1, including roles in vegetative growth, development, and virulence, were evaluated. The corresponding knockout mutants displayed normal development but caused reduced lesion sizes on different plant hosts, indicating that these genes contribute to the virulence of *S. sclerotiorum*. Similar virulence defects have been reported in *C. orbiculare* and *M. oryzae* mutants, but not in *V. dahliae*, suggesting that the tandem peroxidase–oxidase infection paradigm may be conserved in *Colletotrichum, Magnaporthe* and *Sclerotinia*, but not in *Verticillium* species. Although the SsAA5d construct could not be successfully expressed for biochemical characterization, phylogenetic analysis showed that it clusters closely with NhaGalOx, FgrGalOx, NexGalOx, and EfeGalOx, all of which are characterized galactose oxidases [41]. This evolutionary relationship suggests that SsAA5d is likely to function as a galactose oxidase, although its substrate specificity remains to be experimentally validated.

Interestingly, consistent with our phenotypic observations, mining of a public RNA-seq dataset [35] showed that *SsAA5b, SsAA5c, SsAA5d* and *SsPX1* are upregulated during host infection (Figure S4A), whereas *SsAA5a, SsAA5b, SsAA5d* and *SsPX1* exhibit higher expression in older versus young mycelia (Figure S4B). While these trends support our phenotypic findings, comprehensive transcriptomic profiling across controlled developmental and infection stages is required to precisely map their transcriptional dynamics.

Despite our in vitro biochemical characterization, the physiological substrates of these AA5 enzymes remain unknown. They could originate from the fungus, the plant host, or both. Their activities are likely key to the remodeling of cell walls during fungal development and host infection.

In summary, our findings broaden the current understanding of AA5 enzymes by demonstrating their diverse roles in the biology of *S. sclerotiorum*, including sclerotia development, compound appressoria development and virulence. Although the precise mechanisms underlying these functions remain to be elucidated, the contribution of AA5 enzymes to fungal virulence highlights their potential as promising targets for the development of novel disease management strategies. Furthermore, the biochemical characterization of SsAA5c confirms the conserved catalytic activity of fungal AA5_1 enzymes and expands the available biochemical knowledge of this enzyme family. These findings provide a valuable foundation for future studies on the biological functions and catalytic mechanisms of fungal AA5 enzymes and may facilitate the selection and engineering of AA5 biocatalysts for biotechnological applications.

## Figures and Tables

**Table 1:** Michaelis–Menten kinetics parameters for selected substrates for sscle_04g035640, SsAA5c.

Substrate	*K_M_* (mM)	*k_cat_* (s^−1^)	*k_cat_/K_M_* (M^−1^·s^−1^)	pH	Temperature (°C)
D-glyceraldehyde	5.4 ± 0.4	0.9 ± 0.3	172 (91) ^a^	9.0	RT
Methyl glyoxal	n.a	n.a	(64) ^a^	9.0	RT
Glycerol	n.a	n.a	(7) ^a^	9.0	RT

^a^ Values in parentheses correspond to apparent *k_cat_/K_M_* values determined from slopes of linear fit to substrate concentration ranges well below saturation. RT = room temperature (ca. 21 °C), n.a = not applicable. Discrete k_cat_ and K_M_ are not reported for substrates where enzyme saturation was not reached.

## Data Availability

The original contributions presented in this study are included in the article/Supplementary Materials. Further inquiries can be directed to the corresponding author.

## Supplementary Materials

The following supporting information can be downloaded at https://www.mdpi.com/article/10.3390/pathogens15090885/s1; Figure S1. Predicted protein structures and WSC domain sequence alignment of *S. sclerotiorum* AA5_1 members. A. Predicted protein structures of the *S. sclerotiorum* AA5_1 members SsAA5a, SsAA5b, and SsAA5c generated using AlphaFold3. The SignalP region, WSC (wall stress-responsive component) domains, and chitin recognition domains are highlighted in orange, green, and blue, respectively. Amino acid (aa) positions are indicated below each protein schematic. B. Sequence alignments of the WSC (wall stress-responsive component) regions from *S. sclerotiorum* AA5_1 and *S. cerevisiae* WSC1-4 proteins. Conserved cysteine residues characteristic to WSC modules are indicated by arrow. Figure S2. Michaelis–Menten kinetics of SsAA5c on the substrates where activity was seen. Measured in triplicate at room temperature in Glycine NaOH pH 9.0 buffer using the HRP-ABTS coupled assay. Figure S3. Generation and verification of *S. sclerotiorum* AA5 gene knockout mutants. A. Diagram depicting the homologous recombination approach employed for *S. sclerotiorum* AA5 gene deletion. The AA5 coding sequence and the hygromycin resistance cassette (HYG) are represented by brown and green boxes, respectively. The positions of primers used for construct generation and genotype verification are indicated. Scale is shown at the top. B. PCR-based confirmation of *sscle_04g035640* (*SsAA5c)* gene disruption. Genomic DNA from the WT and two independent deletion mutants (*Ssaa5c-1* and *Ssaa5c-2*) were used as template for PCR analysis. Primer pairs 7F + H855R and H855F + 8R were used to confirm homologous integration of the hygromycin resistance cassette, whereas primers 5F + 6R were used to verify deletion of the target gene. Expected amplicon sizes are indicated in brackets. M denotes the DNA size marker. C. PCR-based confirmation of *sscle_08g064880* (*SsAA5a*) gene disruption. Genomic DNA from the WT and two independent deletion mutants (*Ssaa5a-1* and *Ssaa5a-2*) were used as a template for PCR analysis. Primer pairs 7F + H855R and H855F + 8R were used to confirm homologous integration of the hygromycin resistance cassette, whereas primers 5F + 6R were used to verify deletion of the target gene. Expected amplicon sizes are indicated in brackets. M denotes the DNA size marker. D. PCR-based confirmation of *sscle_10g076000* (*SsAA5b*) gene disruption. Genomic DNA from the WT and two independent deletion mutants (*Ssaa5b-1* and *Ssaa5b-2*) were used as template for PCR analysis. Primer pairs 7F + H855R and H855F + 8R were used to confirm homologous integration of the hygromycin resistance cassette, whereas primers 5F + 6R were used to verify deletion of the target gene. Expected amplicon sizes are indicated in brackets. M denotes the DNA size marker. E. PCR-based confirmation of *sscle_15g102370* (*SsAA5d*) gene disruption. Genomic DNA from the WT and two independent deletion mutants (*Ssaa5d-1* and *Ssaa5d-2*) were used as template for PCR analysis. Primer pairs 7F + H855R and H855F + 8R were used to confirm homologous integration of the hygromycin resistance cassette, whereas primers 5F + 6R were used to verify deletion of the target gene. Expected amplicon sizes are indicated in brackets. M denotes the DNA size marker. F. PCR-based confirmation of *sscle_15g102360* (*SsPX1*) gene disruption. Genomic DNA from the WT and two independent deletion mutants (*Sspx1-1* and *Sspx1-2*) were used as template for PCR analysis. Primer pairs 7F + H855R and H855F + 8R were used to confirm homologous integration of the hygromycin resistance cassette, whereas primers 5F + 6R were used to verify deletion of the target gene. Expected amplicon sizes are indicated in brackets. M denotes the DNA size marker. Figure S4. Expression profiles of *SsAA5* genes across developmental and host infection stages. A. Relative transcript abundance across host infection time points. B. Comparative gene expression during vegetative mycelial development. Data derived from public RNA-seq dataset [35]. Table S1. List of recombinant construct sequences. Table S2. List of primers used in this study.
