# Supplementary material for: Copper Radical Oxidases Contribute to Virulence in Sclerotinia sclerotiorum
Source: Pathogens. 2026 Aug 24;15(9):885. doi: 10.3390/pathogens15090885 (PMC13610856; doi:10.3390/pathogens15090885)
Supplement: Supplementary file 1 [file pathogens-15-00885-s001.zip › pathogens-4457064-supplementary.pdf]

Fig S1

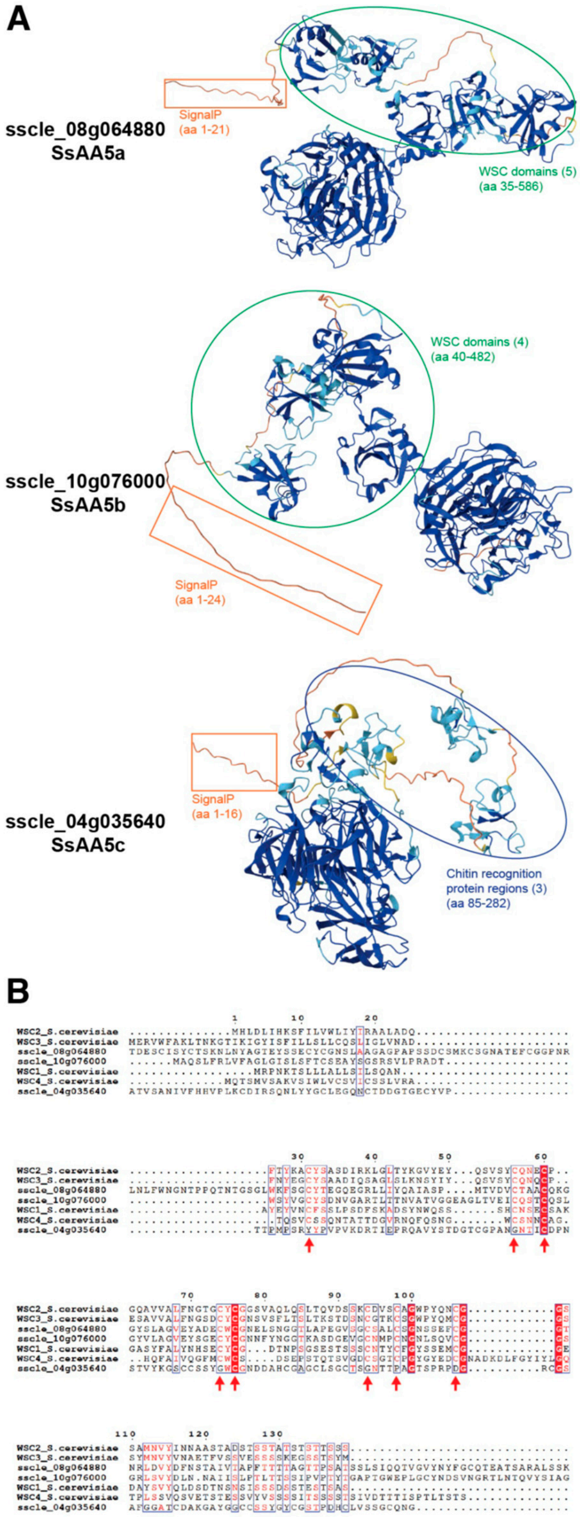

Figure S1. Predicted protein structures and WSC domain sequence alignment of *S. sclerotiorum* AA5_1 members. A. Predicted protein structures of the *S. sclerotiorum* AA5_1 members SsAA5a, SsAA5b, and SsAA5c generated using AlphaFold3. The SignalP region, WSC (wall stress-responsive component) domains, and chitin recognition domains are highlighted in orange, green, and blue, respectively. Amino acid (aa) positions are indicated below each protein schematic. B. Sequence alignments of the WSC (wall stress-responsive component) regions from *S. sclerotiorum* AA5_1 and *S. cerevisiae* WSC1-4 proteins. Conserved cysteine residues characteristic to WSC modules are indicated by arrow.

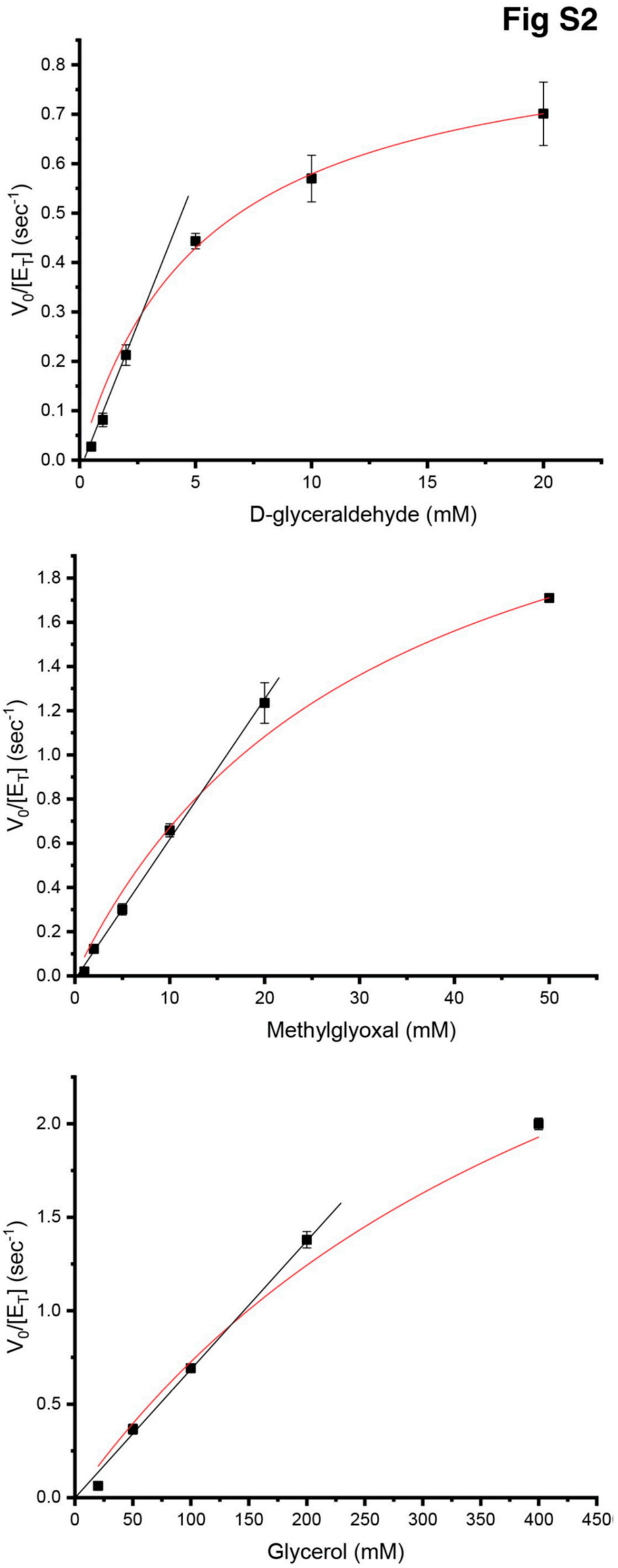

Figure S2. Michaelis–Menten kinetics of SsAA5c on the substrates where activity was seen. Measured in triplicate at room temperature in Glycine NaOH pH 9.0 buffer using the HRP-ABTS coupled assay.

Fig S3

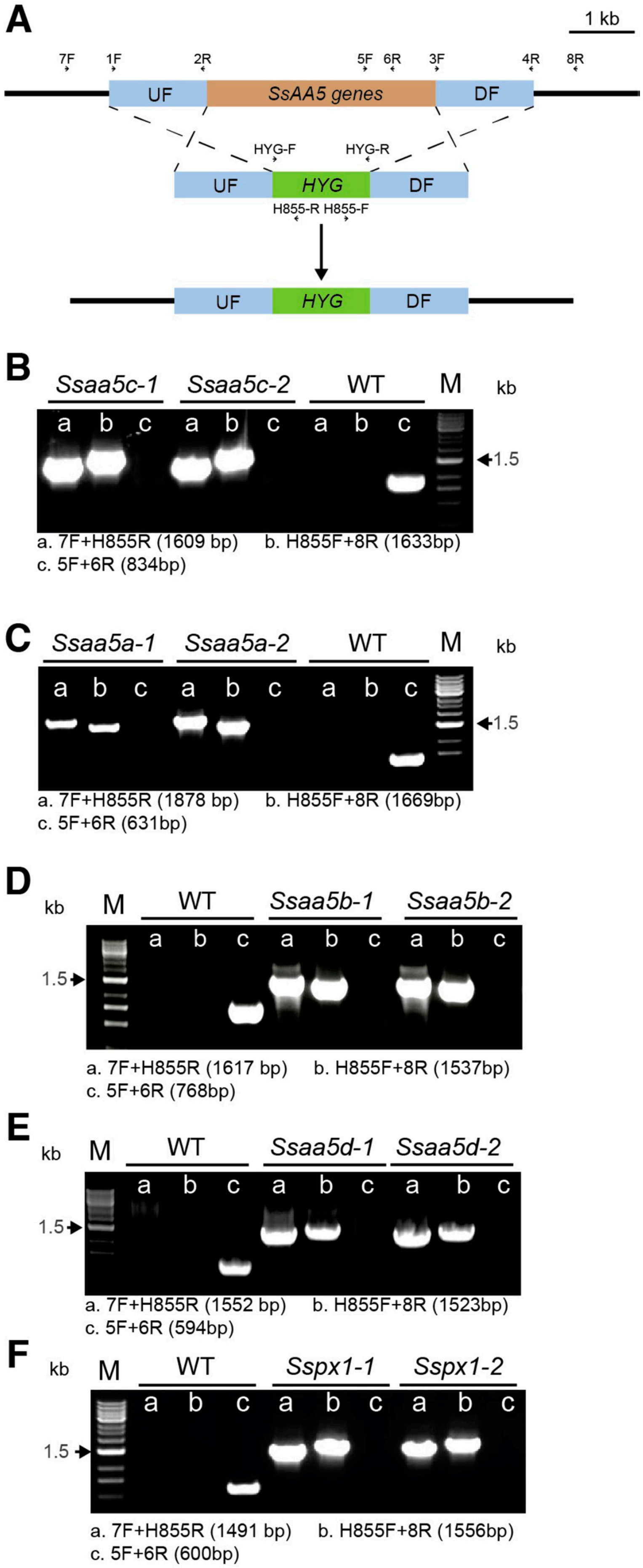
A
1 kb
7F
1F
2R
5F
6R
3F
4R
8R
UF
SsAA5 genes
DF
HYG-F
HYG-R
UF
HYG
DF
H855-R H855-F
UF
HYG
DF
B
Ssaa5c-1
Ssaa5c-2
WT
M
kb
a b c a b c a b c
1.5
a. 7F+H855R (1609 bp) b. H855F+8R (1633bp)
c. 5F+6R (834bp)
C
Ssaa5a-1
Ssaa5a-2
WT
M
kb
a b c a b c a b c
1.5
a. 7F+H855R (1878 bp) b. H855F+8R (1669bp)
c. 5F+6R (631bp)
D
kb
M
WT
Ssaa5b-1
Ssaa5b-2
a b c a b c a b c
1.5
a. 7F+H855R (1617 bp) b. H855F+8R (1537bp)
c. 5F+6R (768bp)
E
kb
M
WT
Ssaa5d-1
Ssaa5d-2
a b c a b c a b c
1.5
a. 7F+H855R (1552 bp) b. H855F+8R (1523bp)
c. 5F+6R (594bp)
F
kb
M
WT
Sspx1-1
Sspx1-2
a b c a b c a b c
1.5
a. 7F+H855R (1491 bp) b. H855F+8R (1556bp)
c. 5F+6R (600bp)

Figure S3. Generation and verification of *S. sclerotiorum* AA5 gene knockout mutants. A. Diagram depicting the homologous recombination approach employed for *S. sclerotiorum* AA5 gene deletion. The AA5 coding sequence and the hygromycin resistance cassette (HYG) are represented by brown and green boxes, respectively. The positions of primers used for construct generation and genotype verification are indicated. Scale is shown at the top. B. PCR-based confirmation of *sscle_04g035640* (*SsAA5c)* gene disruption. Genomic DNA from the WT and two independent deletion mutants (*Ssaa5c-1* and *Ssaa5c-2*) were used as template for PCR analysis. Primer pairs 7F + H855R and H855F + 8R were used to confirm homologous integration of the hygromycin resistance cassette, whereas primers 5F + 6R were used to verify deletion of the target gene. Expected amplicon sizes are indicated in brackets. M denotes the DNA size marker. C. PCR-based confirmation of *sscle_08g064880* (*SsAA5a*) gene disruption. Genomic DNA from the WT and two independent deletion mutants (*Ssaa5a-1* and *Ssaa5a-2*) were used as a template for PCR analysis. Primer pairs 7F + H855R and H855F + 8R were used to confirm homologous integration of the hygromycin resistance cassette, whereas primers 5F + 6R were used to verify deletion of the target gene. Expected amplicon sizes are indicated in brackets. M denotes the DNA size marker. D. PCR-based confirmation of *sscle_10g076000* (*SsAA5b*) gene disruption. Genomic DNA from the WT and two independent deletion mutants (*Ssaa5b-1* and *Ssaa5b-2*) were used as template for PCR analysis. Primer pairs 7F + H855R and H855F + 8R were used to confirm homologous integration of the hygromycin resistance cassette, whereas primers 5F + 6R were used to verify deletion of the target gene. Expected amplicon sizes are indicated in brackets. M denotes the DNA size marker. E. PCR-based confirmation of *sscle_15g102370* (*SsAA5d*) gene disruption. Genomic DNA from the WT and two independent deletion mutants (*Ssaa5d-1* and *Ssaa5d-2*) were used as template for PCR analysis. Primer pairs 7F + H855R and H855F + 8R were used to confirm homologous integration of the hygromycin resistance cassette, whereas primers 5F + 6R were used to verify deletion of the target gene. Expected amplicon sizes are indicated in brackets. M denotes the DNA size marker. F. PCR-based confirmation of *sscle_15g102360* (*SsPX1*) gene disruption. Genomic DNA from the WT and two independent deletion mutants (*Sspx1-1* and *Sspx1-2*) were used as template for PCR analysis. Primer pairs 7F + H855R and H855F + 8R were used to confirm homologous integration of the hygromycin resistance cassette, whereas primers 5F + 6R were used to verify deletion of the target gene. Expected amplicon sizes are indicated in brackets. M denotes the DNA size marker.

Fig S4

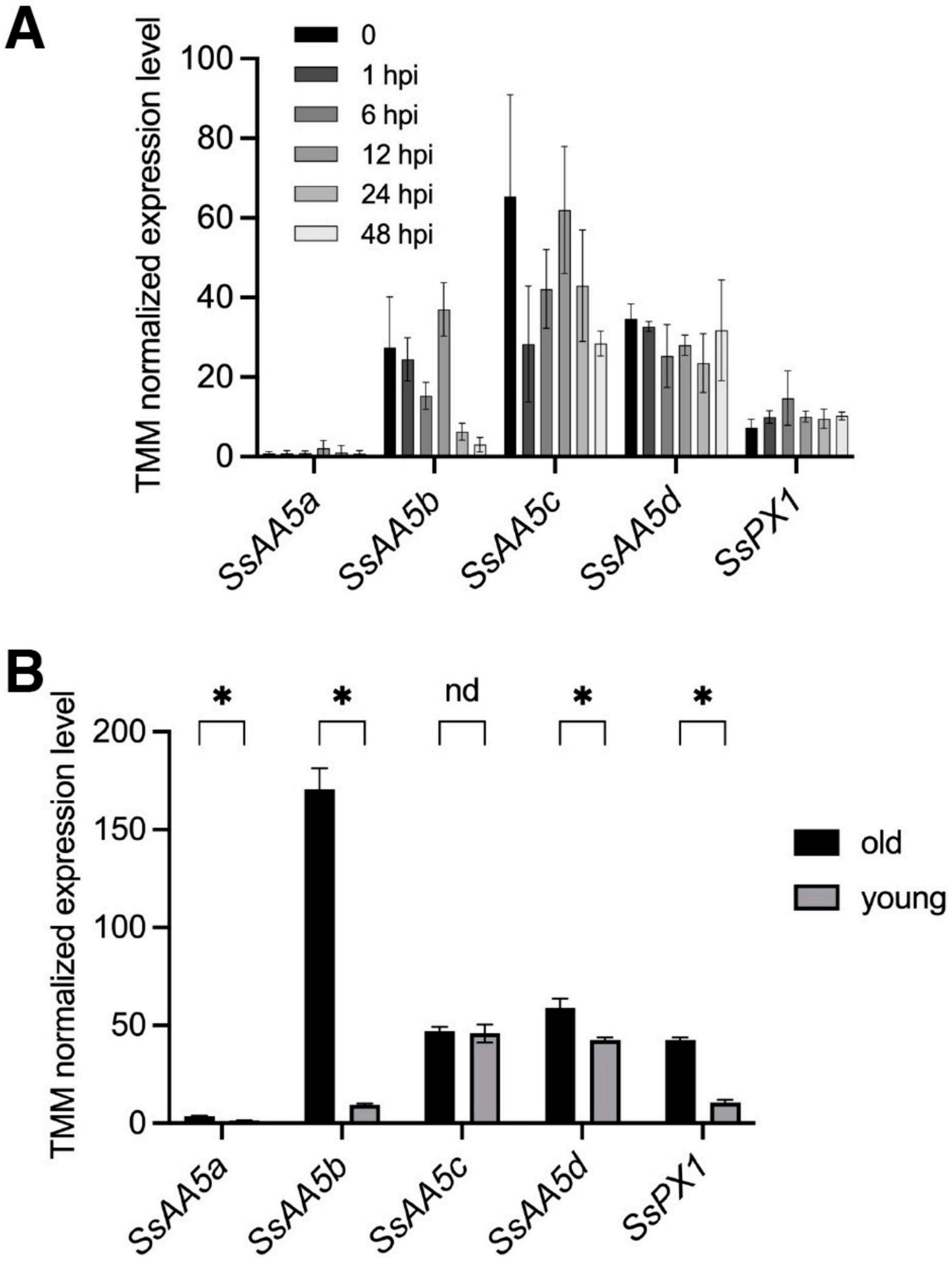

Figure S4. Expression profiles of *SsAA5* genes across developmental and host infection stages. A. Relative transcript abundance across host infection time points. B. Comparative gene expression during vegetative mycelial development. Data derived from public RNA-seq dataset [35].

Table S1. List of recombinant construct sequences.

Gene ID	Optimized sequence based on *Pichia* codon
sscle_04g035640 (*SsAA5c*)	**CGGAATTC**ATGAAGGCTACTTTTTTGTCCTTGGGTTTTATCGCTACTGTTTCCG<br>CTAACATCGTTTTTCATCATGTTCCATTGAAGTGTGACATCAGATCCCAGAACT<br>TGTACTACGGTTGTTTGGAAGGTCAGAACTGTACTGACGACGGTACTGGTGA<br>ATGTTACGTTCCAACTACTCCAATGCCATCCAGATACTACCCAGTTCCAGTTAA<br>GGACAGAACTATCGAACCAAGACAGGCTGTTTACTCCACTGACGGTACTTGT<br>GGTCCAGCTAACGGTAACACTATCTGTGACCCAAACTCCACTGTTTACAAGGG<br>TTCCTGTTGTTCCTCCTACGGTTGGTGTGGTAACGACGACGCTCATTGTGGT<br>GCTGGTTGTTTGTCCGGTTGTACTTCCGGTAACACTACTCCAGCTGGTACTTC<br>CCCAAGACCAGACGGTAGATGTGGTTCCGCTTTTGGTGGTGCTACTTGTGAC<br>GCTAAGGGTGCTTACGGTGGTTGTTGTTCCTCCTACGGTTACTGTGGTTCCAC<br>TCCAGACCATTGTTTGGTTTCCTCCGGTTGTCAGAACGGTTGTACTGACGCTA<br>AGTCCACTACTGTTGCTCAGTCCACTACTACTGCTGCTGGTGGTAACGCTGCT<br>CCATCCTCCACTTCCCAGGAACCAGTTATCGCTCCAGTTACTTCCACTTTGTC<br>CCCAGCTGCTGCTTCCAACGCTCCAGTTACTAAGGACGGTTCCTGTGGTACT<br>GCTAACGGTGGTACTGTTTGTGGTAACTGGCCAAACGGTAACTGTTGTTCCAT<br>GTACGGTTTTTGTGGTAACACTAACGCTCATTGTGGTGCTGGTTGTCAGTCCG<br>GTAACTGTTTGAACGGTCCAGTTATCCCAGCTCCAGGTGCTTCCCCAGCTCC<br>AGCTGCTCCAGTTGGTGGTGCTTTTAACATCGTTGGTCAGTCCGGTGTTCCA<br>GCTATGCATGCTGCTTTGATGCAGAACGGTAGAGTTATGTTTTTGGACAAGTT<br>GGAAAACTACACTCAGTTGAGATTGCCAAACGGTAACTACGCTATGTCCTCCG<br>AATACGACCCAGCTACTAACGGTGTTGCTGCTCCATTGGCTTACAAGACTAAC<br>GCTTTTTGTTCCGGTGGTACTTTTTTGGCTGACGGTAGAGTTGTTTCCTTGGG<br>TGGTAACGCTCCATTGTCCTGGTTGGACCCAACTATCGGTGACGGTTTTACTG<br>CTATCAGATACTTGGAAAGATCCTCCACTGACGCTTCCTTGACTGGTAAGGAC<br>TGGTCCGAACCAGGTAACAAGTTGGCTTCCGCTAGATGGTACGCTACTGCTC<br>AGACTATGGCTGACGGTACTATCTTTGTTGCTTCCGGTTCCTTGAACGGTTTG<br>GACCCAACTGTTAACACTAACAACAACCCAACTTACGAAATCTTGTCCCCAAC<br>TGCTGTTTCCCAGGGTAAGAACATCGACTTGGAAATCTTGGTTAAGAACCAGC<br>CATACTACATGTACCCATTTGTTCATTTGTTGAACGACGGTAACTTGTTTATCTT<br>TGTTTCCAAGTCCTCCCAGATCTTTAACGTTGGTACTAACTCCATCGTTAAGGA<br>ATTGCCAGAATTGGCTGGTGACTACAGAACTTACCCAAACACTGGTGGTTCC<br>GTTTTGTTGCCATTGTCCTCCGCTAACAACTGGAACCCAGACATCGTTATCTG<br>TGGTGGTGGTGCTTACCAGGACATCACTTCCCCAACTGAACCATCCTGTGGT<br>AGAATCCAGCCATTGTCCGCTAACCCAACTTGGGAATTGGACTCCATGCCAGA<br>AGGTAGAGGTATGGTTGAAGGTAACTTGTTGCCAGACGGTACTGTTATCTGGT<br>TGAACGGTGGTAACTTGGGTGCTCAGGGTTTTGGTTTGGCTAAGGCTCCAAC<br>TTTGGAAGCTTTGTTGTACGACCCAACTAAGGCTAAGGGTCAGAGATTTTCCA<br>CTTTGGCTACTTCCACTATCCCAAGATTGTACCATTCCGTTTCCTTGTTGTTGT<br>TGGACGGTACTTTGATGGTTGCTGGTTCCAACCCAGTTGAAATGCCAAAGTTG<br>AAGCCAGACGCTGCTGACGAATTTGTTACTGAATTTAGAGTTGAAAACTACGT<br>TCCACCATACTTGTCCGGTGACAACGCTAACAAGAGACCAACTAACGTTAAGT<br>TGTCCTCCGGTTCCTTTAAGGCTGACGGTTCCACTTTGGACGTTACTTTTAAC<br>TGTCCAGCTGGTGCTAAGGCTGTTACTGTTACTTTGTACCATGGTGGTTTTGT<br>TACTCATTCCGTTCATATGGGTCATAGAATGTTGCATTTGGACAACACTGGTTT<br>TGTTGCTGGTGCTACTCAGCAGAAGTTGACTGTTACTAGACCACCAAACAACA<br>ACGTTGCTCCACCAGGTCCATACGTTGTTTACATCTTGGTTGACGGTATCCCA<br>GCTATCGGTCAGTTTGTTACTGTTGG**TCTAGAGC**
sscle_15g102370 (*SsAA5d*)	**CGGAATTC**ATGAAGTCCCAGTTGACTAGATTGTTGTTGGGTGTTTCCTTTGGT<br>GGTGTTAACGCTGTTAGAAACTTTATGACTGAGGCTGCTAACTCCCAGAGAAT<br>CTCCGGTTACGGTCCAGTTGACAAGCCATCCTGGGAGCCAACTTTTATCGAC<br>GAGTCCCCACCATACCAGGGTATCAGAATCCCAAGACAGGAGTGGAAGTTGG<br>AGTGTTCCTCCGCTGAGACTTCCTTTCCATGTCAGAACGCTATCGACGGTACT

	TCCACTTCCTCCTGGAGATCCCAGTTGGGTTCCGAGTCCCATTCCATCATCGT<br>TGACTTGAACAAGCCACATGCTGTTAACGGTTTGGTTATCTTGCCACCAATCG<br>ACTCCGAGTCCAAGGGTTTGATCACTCAGCATGAGATCTACACTTCCAACGAC<br>AAGAAGGACTGGAAGTCCGTTGCTTACGGTATGTGGCCAAACACTAACAGAC<br>AGAGATTGTCCGCTTTTGAGCCAACTTCCGCTAGATACGTTAAGTTGAACGTT<br>TCCACTGGTGTTGGTGGTCCATCCTGGATCGGTATCTCCGAGATCAACATCTA<br>CGCTACTTTGTACACTATCCCACAGGACCCAAAGTTGGGTGTTTGGGGTCCA<br>ACTATCGACTTTCCAGTTGTTCCAGTTGCTGGTGCTCAGGAGGCTTCCGGTAA<br>GATCGTTTTGTGGTCCTCCTGGGCTTCCGACAGATTTAAGTCCACTCCAGGT<br>GGTAAGACTGCTATGGCTAGATGGGACCCATCCACTTCCGAGGTTTCCGAGA<br>GAATCGTTACTGACACTGGTCATGACATGTTTTGTCCAGGTATCTCCATCGAC<br>GGTACTGGTGTTATGGTTGTTACTGGTGGTAACGACGCTTCCGTTACTTCCTT<br>GTACGACGCTTCCACTGACCAGTGGACTAAGGGTCCAGCTATGTCCTTGAGA<br>AGAGGTTACCAGTCCTCCACTACTTTGTCCGACGGTAGAGTTTTTGTTATCGG<br>TGGTTCCTGGCATGGTACTGCTAGATTGCCAAAGGACGGTGAGGTTTACGAC<br>CCAAACAAGAAGACTTGGTCCATGTTGCCAGGTGCTACTGTTGAGCAGATGT<br>TGACTGAGGACATGGAGGGTCCATGGAGAGCTGACAACCATGGTTGGTTGTT<br>TGGTTGGAAGAACGGTTCCGTTTTTCAGGCTGGTCCATCCAGAGCTATGAAC<br>TGGTACTTTACTGACGAGAACGGTTCCGTTCAGGGTGCTGGTCCAAGATTGA<br>ACGACGCTGACTCCATGTCCGGTAACGCTGTTATGTTTGACGCTACTGAGGG<br>TAAGATCTTGACTATCGGTGGTTCCCCAGACTACGACGCTTCCTTTGCTACTT<br>CCAACGCTCATGTTATCACTTTGGGTGAGCCAAGAGAGCATGTTGACGTTAAG<br>CCAGCTGGTATCGGTGGTAAGATGCATTCCAAGAGAGTTTTTCATTCCTCCGT<br>TGTTTTGCCAGACGGTACTGTTTTTATCGCTGGTGGTCAGACTTTTGGTGTTG<br>CTTTTAACGAGGAGAACGTTCAGTTTGTTCCAGAGTTGTACGACCCAAAGACT<br>AACACTTTTACTGAGTTGTTGTCCAACAACGTTATCAGAGTTTACCATACTTTG<br>TCCATCTTGTTGGCTGACGGTAGAGTTTTGAACGCTGGTGGTGGTTTGTGTG<br>GTGACTGTTCCGCTAACCATTACGACGGTCAGATCTTTACTCCACCATACTTGT<br>TGACTGAGGAGGGTAAGTTGAGAGACAGACCAGAGATCAGATCCACTGTTCC<br>ATCCGAGTTGAACGTTGGTGACGTTTTTGAGTTGAAGGCTTCCGTTCCAATCC<br>ATTCCGCTTCCTTGATCAGAATCGGTTCCGCTACTCATACTGTTAACACTGACC<br>AGAGAAGAGTTCCATTGCCATTGTCCAGATCCGGTTTGCATAGAAACGGTTAC<br>ACTGCTAGATTGCCAACTTCCTCCGGTATCTTGATCCCAGGTTACTGGATGTT<br>GTTTGTTATGGACGAGTACGGTACTCCATCCAGAGCTGAGGTTATCTTTATCAA<br>CACTCCAAAGGCTATCGACTCCTACGGTGAGGAGCAGTCCCAGTTGGGTTTG<br>TTGGAGTCCTACTGGCAGTCCTGGAAGCCAACTTTGATGGCTCAGTTTTCCA<br>GACCAGGTAGAGAGTTGATCGG**TCTAGAGC**

Restrict enzymes are in bold.

Table S2. List of primers used in this study.

Primer name	DNA sequence (5’ to 3’)	Purpose
SsAA5c-1F	GAAGCTTAAAGGTGGTGAACC	*SsAA5c* knockout
SsAA5c-2R	cgggtaccgagctctttgaaggttgtgGCCTTCATGGCTTCTAAAGG	
SsAA5c-3F	gccgaccgggatccacttaacgttacGGAATACGGCGTTATGTGTG	
SsAA5c-4R	GTGCGAGACTCATCATAGTCG	
SsAA5c-5F	CTACATGTACCCTTTTGTCC	
SsAA5c-6R	CAACTTTTGCTGTGTTGCAC	
SsAA5c-7F	GCCTTTCCAATTTCTGGATG	
SsAA5c-8R	GCCGGAGTATGCAATCTCTC	
SsAA5a-1F	GTGGTAGCTTCGTATATCC	*SsAA5a* knockout
SsAA5a-2R	cgggtaccgagctctttgaaggttgtgGTCAATGTATTCTCCCTCG	
SsAA5a-3F	gccgaccgggatccacttaacgttacGAGTTTGGGTACGTATTGGTG	

SsAA5a-4R	GTATCCGTGCTTTCCACTC	
SsAA5a-5F	GAAACTGGCAGTAATGCGC	
SsAA5a-6R	GGATAAGTGTTGTGCCGTC	
SsAA5a-7F	GCAGAAGACGAGAGAAATTG	
SsAA5a-8R	CTCATATTCCAGGCATGCAC	
SsAA5b-1F	GAGTATCATTATCTCGGGTAG	*SsAA5b* knockout
SsAA5b-2R	cgggtaccgagctctttgaaggttgtgGTCGCTCGATGATATACAGG	
SsAA5b-3F	gccgaccgggatccacttaacgttacGCTGTCTTTTTGGGCAATG	
SsAA5b-4R	GAGATGACTTTTTCGAGCC	
SsAA5b-5F	CAAAGTGGACCATTGAGCGC	
SsAA5b-6R	CTGGGAAATTAGGCCACTCG	
SsAA5b-7F	GAAGGCGGGTCCTAATTTTAC	
SsAA5b-8R	CACGTTACGGCTGTTATTCG	
SsAA5d-1F	ACAGCTCTGCCCAAAGAAGA	*SsAA5d* knockout
SsAA5d-2R	cgactctagaggatcccggTCACGCCTCCAAAAGAGACT	
SsAA5d-3F	gttggtgtcgatgtcagctcAGACCTTTGGGGTTGCTTTC	
SsAA5d-4R	GGTTTGCTTGGTGTTGAGGT	
SsAA5d-5F	CACCAGCAACGACAAGAAGG	
SsAA5d-6R	CCACCAATGACGAAAACACG	
SsAA5d-7F	CATCCCAATGATTCCTTTCTC	
SsAA5d-8R	GAAGTTTCACTGTCGTATTCG	
SsPX1-1F	TGAAGAGTGTGTCGCCTGTC	*SsPX1* knockout
SsPX1-2R	cgactctagaggatcccggTTGGAATCTTTTGGGCAGTC	
SsPX1-3F	gttggtgtcgatgtcagctcCTTCTCGCCGAGTATCAAGG	
SsPX1-4R	TGGGATTTAACCCACACCAT	
SsPX1-5F	AAAAGGGCAAAGAGCAGTGA	
SsPX1-6R	TGTTGGAACGGAAAAGGTTC	
SsPX1-7F	GGAACGTCGATCATAACAAG	
SsPX1-8R	GTGCGTGTCCGCTAAAATTG	
HYG-F	CACAACCTTCAAAGAGCTCGGTACCCG	*HYG* gene amplification
HYG-R	GTAACGTTAAGTGGATCCCGGTCGGC	
Hygro-F	CCGGGATCCTCTAGAGTCG	
Hygro-R	GAGCTGACATCGACACCAAC	
H855F	GTCGATGCGACGCAATCGT	Check *HYG gene insertion*
H855R	GAACCATCTTGTCAAACGAC	
